# Protective Effects of Anethole in *Foeniculum vulgare* Mill. Seed Ethanol Extract on Hypoxia/Reoxygenation Injury in H9C2 Heart Myoblast Cells

**DOI:** 10.3390/antiox13101161

**Published:** 2024-09-25

**Authors:** Jeong Won Seo, Sarmin Ummey Habiba, Yeasmin Akter Munni, Ho Jin Choi, Asma Aktar, Kishor Mazumder, Deuk-Young Nah, In-Jun Yang, Il Soo Moon

**Affiliations:** 1 Division of Cardiology, Department of Internal Medicine, College of Medicine, Dongguk University, Gyeongju 38066, Republic of Korea; thebboys@hanmail.net (J.W.S.); ptca@dongguk.ac.kr (D.-Y.N.); 2 Department of Anatomy, College of Medicine, Dongguk University, Gyeongju 38066, Republic of Korea; sarmin.ummey.habiba07@gmail.com (S.U.H.); yeasminakteracce@gmail.com (Y.A.M.); chjack@naver.com (H.J.C.); 3 Department of Physiology, College of Korean Medicine, Dongguk University, Gyeongju 38066, Republic of Korea; injuny@dongguk.ac.kr; 4 Medical Institute of Dongguk University, Gyeongju 38066, Republic of Korea; 5 Department of Pharmacy, Jashore University of Science and Technology, Jashore 7408, Bangladesh; a.aktar@just.edu.bd (A.A.); kmazumder@just.edu.bd (K.M.)

**Keywords:** ADMET, boiled-egg model, DNA double-strand break damage, *Foeniculum vulgare* Mill., GC-MS, H9C2, mitochondrial membrane potential, nuclear condensation, ROS

## Abstract

Background: Active compounds from plants and herbs are increasingly incorporated into modern medical systems to address cardiovascular diseases (CVDs). *Foeniculum vulgare* Mill., commonly known as fennel, is an aromatic medicinal plant and culinary herb that is popular worldwide. Methods: Protective effects against cellular damage were assessed in the H9C2 cardiomyocyte hypoxia/reoxygenation (H/R) experimental model. The identities of phytochemicals in FVSE were determined by GC-MS analysis. The phytochemical’s potential for nutrients and pharmacokinetic properties was assessed by ADMET analysis. Results: GC-MS analysis of the ethanol extracts of *F. vulgare* identified 41 bioactive compounds, with four prominent ones: anethole, 1-(4-methoxyphenyl)-2-propanone, ethoxydimethylphenylsilane, and para-anisaldehyde diethyl acetal. Among these, anethole stands out due to its potential for nutrients and pharmacokinetic properties assessed by ADMET analysis, such as bioavailability, lipophilicity, flexibility, and compliance with Lipinski’s Rule of Five. In the H/R injury model of H9C2 heart myoblast cells, FVSE and anethole suppressed H/R-induced reactive oxygen species (ROS) generation, DNA double-strand break damage, nuclear condensation, and the dissipation of mitochondrial membrane potential (ΔΨm). Conclusions: These findings highlight the therapeutic potential of FVSE and its prominent component, anethole, in the treatment of CVDs, particularly those associated with hypoxia-induced damage.

## 1. Introduction

Cardiovascular diseases (CVDs) are the leading cause of death worldwide, claiming approximately 17.9 million lives annually (WHO, https://www.who.int/health-topics/cardiovascular-diseases#tab=tab_1, accessed in 10 June 2024). In many CVD cases, the supply of oxygen and nutrients is reduced or completely stopped. Various mechanisms, such as enhanced oxygen transport and delivery along with increased blood flow, are activated to counteract the hypoxic condition. If these compensatory mechanisms are inadequate to resolve hypoxia, irreversible damage may ensue. Therefore, hypoxia is a key factor in the development and progression of CVDs [1]. Research into CVDs has been extensive, with a focus on discovering new therapeutic agents that can mitigate the detrimental effects of hypoxia [2]. Hypoxia detrimentally affects the integrity of cardiomyocytes, which plays a significant role in the development of CVDs, such as atherosclerosis, pulmonary arterial hypertension, vascular remodeling, and heart failure [3,4,5,6]. Therefore, the creation of effective treatments that offer cardioprotection in hypoxic conditions is crucial.

Traditional treatments for CVDs are often expensive and may have undesirable side effects, leading to the exploration of alternative therapies, particularly those derived from medicinal plants [7]. In this context, bioactive compounds from natural products have become particularly significant for the prevention and management of CVDs. Lifestyle interventions, such as healthy diets, are strongly integrated into international health policies. Specifically, plant-based and Mediterranean diets are recommended as the most beneficial for health [8,9], as they include a variety of components rich in nutrients and bioactive phytochemicals, like flavonoids.

In recent years, natural products have been recognized as potential sources for novel cardio-protective agents [2,4]. Purified extracts and active compounds from plants and herbs are increasingly incorporated into modern medical systems to address conditions such as arrhythmia, cerebral insufficiency, venous insufficiency, atherosclerosis, congestive heart failure, angina pectoris, and systolic hypertension. Notable plants with a history of treating CVDs include *Ginkgo biloba*, *Daucus carota*, *Salvia miltiorrhiza*, *Amaranthus viridis*, *Nerium oleander*, *Andrographis paniculata*, *Terminalia arjuna*, *Tinospora cordifolia*, *Mucuna pruriens*, *Picrorhiza kurroa*, *Bombax ceiba*, *Hydrocotyle asiatica*, and more [10]. These natural products are rich in phytochemicals with cardioprotective properties, including terpenoids, phenols, iridoids, saponins, flavonoids, glycosidic derivatives, alkaloids, polyphenols, plant sterols, and sulfur compounds [11,12].

Cardioprotective phytochemicals have been identified, including silymarin, cyclovirobuxine D, withanolides, curcumin, berberine, tilianin, baicalin, resveratrol, naringenin, allicin, and myricitrin [11]. Notoginsenoside R1, along with ginsenosides Rg1, Rg3, Rb1, and R1 from Panax notoginseng, has shown potential for preventing thrombosis, protecting myocardial cells from ischemia, dilating peripheral arteries, and preventing ischemic brain damage. Curcumin from Curcuma longa rhizomes has been effective in preventing aortic aneurysms, myocardial infarctions, strokes, and atherosclerosis. Additionally, allicin, an active component in garlic, and quercetin, a flavonoid present in various fruits and vegetables, have been linked to reduced arterial clot formation, thereby lowering the risks of heart attacks and strokes [11,12].

*F. vulgare*, commonly known as fennel, is an aromatic medicinal plant and culinary herb that is popular worldwide [13]. *F. vulgare* is renowned for its rich phytochemical composition, which includes flavonoids, phenolic compounds, and essential oils [14]. This plant exhibits a spectrum of bioactivities, ranging from its well-known antioxidative properties to its benefits of digestive support, blood pressure modulation, cognitive function enhancement, and immune system fortification, which are relevant in the context of hypoxia-related cardiac damage [13].

The present research aims to systematically evaluate the potential of *F. vulgare* seed ethanol extracts (FVSE) in mitigating hypoxia/reoxygenation (H/R)-induced cardiomyocyte damage. We employed a two-pronged approach integrating an in vitro H/R experimental model with an assessment of nutrient potential and pharmacokinetic properties through ADMET analysis [14], including bioavailability, lipophilicity, flexibility, and compliance with Lipinski’s Rule of Five [15,16]. This investigation provides insights into FVSE and its components’ cardioprotective mechanisms, contributing to the broader field of cardioprotective natural therapeutics and offering potentially groundbreaking insights into their application against cardiac dysfunctions.

## 2. Materials and Methods

### 2.1. Chemicals and Reagents

Anethole (purity >98.0% GC) was obtained from Tokyo Chemical Industry Co., Ltd. (TCI, CAS: 4180-23-8, Kita-ku, Tokyo, Japan), dissolved in dimethyl sulfoxide (DMSO) to achieve a stock concentration of 50 mM, and stored at −20 °C for future use. Unless noted otherwise, cell culture media and supplements were purchased from Invitrogen (Carlsbad, CA, USA).

### 2.2. Preparation of Extracts

*F. vulgare* Mill. seeds were sourced from a farm in Gyeongsang Province, Republic of Korea. The GPS coordinates for the location are approximately 36.04622° N latitude and 129.05347° E longitude. (GPS Coordinates; https://www.gps-coordinates.net/, accessed in 10 June 2024). The fennel samples underwent a rigorous extraction protocol. Initially, the *F. vulgare* seeds were cleaned with distilled water and then air-dried at ambient temperature to preserve their phytochemical integrity. The seeds were powdered using a pottery and mortar set, and two grams of this powder were soaked in 95% ethanol (1:50 *w*/*v*). The suspension was agitated at 200 revolutions per minute (RPM) using an orbital shaker (VS-202D, Vision Scientific Co. Ltd., Seoul, Republic of Korea) at room temperature for 48 h in a light-excluded environment to protect the sensitive components. Post-agitation, the mixture was filtered through sterile cotton to remove particulate matter, collecting a liquid known as the supernatant. This supernatant was then concentrated under a nitrogen gas stream in vacuum conditions to completely evaporate the solvent. The resultant concentrated extract (FVSE, *F. vulgare* Mill. seed ethanol extracts) was reconstituted in DMSO to achieve a final concentration of 8 mg/mL, prepared in foil-wrapped vials, and stored at −20 °C to ensure its stability for subsequent experimental analysis. This extract, properly cataloged, is available (voucher specimen no. MOONIS-2024-FVSE) for study at Il Soo Moon’s Lab (Dongguk University College of Medicine, Department of Anatomy, Republic of Korea).

### 2.3. H9C2 Cell Culture

Rat H9C2 cells, obtained from the Korean Cell Line Bank (Seoul, Republic of Korea), were cultured in DMEM medium supplemented with 10% fetal bovine serum and 1% penicillin–streptomycin. For morphological and viability studies, cells were plated at a density of 3.0 × 10^4^ cells/cm^2^ on 12 mm glass coverslips coated with poly-DL-lysine (PDL, Sigma-Aldrich, CAS 61686-25-7, St. Louis, MO, USA) in 24-well plates. The cultures were maintained at 37 °C in an atmosphere of 95% air and 5% CO_2_.

### 2.4. Hypoxia/Reoxygenation (H/R)

H9C2 cells were cultured at a concentration of 3.0 × 10^4^ cells/cm^2^ and initially received one of three treatments as the cells reached 70–80% confluence: Control, H/R without seed extract (FVSE) [H/R (−FVSE)], or 50 µg/mL of FVSE [H/R (+FVSE)] for two hours. For hypoxia/reoxygenation (H/R) treatment, cells on coverslips were placed in a hypoxic chamber (Modular Incubator Chamber MIC-101; Billups-Rothenberg Inc., Del Mar, CA, USA) with 94% N_2_, 5% CO_2_, and 1% O_2_ at 37 °C for 8 h. Afterwards, the cells were moved back to a normoxic environment to reoxygenate with 95% air and 5% CO_2_ at 37 °C for 16 h to prepare for subsequent experiments.

### 2.5. Cell Viability

Dead cells detach over time from the substrate coverslip. Therefore, cell viability was assessed in two ways. First, the number of cells remaining attached was counted. Second, live and dead cells were differentiated by staining with trypan blue, which was excluded from live cells. This analysis shows the ratio of live to dead cells at the time of trypan blue staining. For this purpose, cells were stained with 0.4% (*w*/*v*) trypan blue dye for 15–20 min at room temperature. After staining, the coverslips were thoroughly rinsed with phosphate-buffered saline (PBS; Invitrogen) to eliminate any residual dye. Only cells with compromised membrane integrity, which absorbed the dye, appeared blue under a phase-contrast microscope. Viability was determined by the proportion of unstained (viable) cells relative to the total number of cells, with results normalized to control samples not subjected to H/R stress. The cell viability for control groups under normoxic conditions was set at 95%, and the viability of experimental groups was expressed as a percentage relative to this baseline.

### 2.6. Measurement of Intracellular Reactive Oxygen Species (ROS) Levels

The production of ROS related to mitochondrial dysfunction was investigated using the DCFDA staining method [17]. Control (-H/R), [H/R (−FVSE)], and [H/R (+FVSE)]-treated H9C2 cell cultures were exposed to 10 µM DCFDA under dark conditions for 15 min at 37 °C to ensure stable environmental conditions. Following this incubation, the cultures were washed with 1x PBS to eliminate residual dye. The cells were then examined under a fluorescence microscope to detect and quantify ROS-positive cells using the FL-1 green channel.

### 2.7. Determination of Mitochondrial Membrane Potential

The mitochondrial membrane potential (ΔΨm) was measured using 5,5′,6,6′-tetrachloro-1,1′,3,3′-tetraethyl benzimidazolyl carbocyanine iodide (JC-1). H9C2 cells were treated with 1 μg/mL JC-1 dye (Molecular Probes) and incubated at 37 °C for 30 min to allow the dye to interact with the mitochondrial membranes. After incubation, the cells were washed twice with freshly warmed media to remove any unbound dye and observed under a fluorescence microscope. The red-to-green fluorescence intensity ratio was analyzed using ImageJ software (version 1.45; National Institutes of Health [NIH], Bethesda, MD, USA) to quantitatively evaluate the ΔΨm levels [18].

### 2.8. Nuclear Condensation

H9C2 cells were subjected to hypoxia for 8 h followed by 16 h of reoxygenation. After the reoxygenation period, the cells were rinsed with PBS and fixed using a sequential fixation method involving 4% paraformaldehyde for 10 min at room temperature, followed by methanol fixation [19]. For nuclear visualization, the cells were stained with DAPI at a concentration of 1 mg/mL and then imaged using a fluorescence microscope. The extent of nuclear condensation was quantitatively assessed by counting 200 to 300 cells from each sample to determine the proportion of cells with condensed chromatin.

### 2.9. Immunocytochemistry 

After transitioning to hypoxia for 8 h followed by 16 h of reoxygenation, the cells were briefly rinsed with 1× PBS and fixed using a sequential paraformaldehyde/methanol fixation procedure, as described by [19]. For the immunostaining procedure, the coverslips were incubated overnight at 4 °C with a rabbit polyclonal anti-phospho-H2AX antibody (1:500 dilution, Millipore, Burlington, MA, USA). Subsequently, the coverslips were incubated with secondary antibodies: Alexa Fluor 568-conjugated goat anti-rabbit IgG (1:1000 dilution, Molecular Probes). Following the immunostaining procedure, the cells were washed and mounted on slides [19].

### 2.10. Gas Chromatography-Mass Spectrometry (GC-MS) Analysis

The GC-MS analysis was conducted using a full scan mode, scanning across a wide range of mass-to-charge ratios (m/z) ranging from 30 to 500. The mass spectra obtained were then compared with the NIST Database to identify the compounds. To analyze the sample, a meticulous sample pre-treatment process was followed. A 0.1 g portion of the sample was dissolved in 10 mL of DMSO and agitated in a shaking incubator set at 250 rpm and 30 °C for 4 h. After incubation, the sample was filtered through a 0.45 µm PTFE filter to remove any particulates. The filtered sample was then analyzed using an Agilent 7890A gas chromatograph coupled with a 5975C mass spectrometer at the Korea Polymer Testing & Research Institute (Koptri), Ltd. (Seoul 02633, Republic of Korea). Separation was achieved on a DB-5MS UI column (30 m length, 0.25 mm internal diameter, and 0.25 µm film thickness). The inlet temperature was maintained at 220 °C with a 1 μL injection volume, and the MS source temperature was set to 230 °C. A solvent delay of 3 min was incorporated, and the split ratio was 10:1 with a carrier gas flow rate of 1.0 mL/min in EI mode. The oven temperature program initiated at 40 °C, was held for 5 min, followed by a ramp of 10 °C/min to a final temperature of 250 °C, maintained for 5 min. The chemical identities of peaks were determined by screening the NIST12.0 spectral library (https://www.nist.gov/pml/atomic-spectra-database) using the chemical’s fragmentation patterns.

### 2.11. In Silico Pharmacokinetics Analysis 

The molecular structures of the identified phytochemicals were obtained from the PubChem database (https://pubchem.ncbi.nlm.nih.gov/, accessed in 15 June 2024). In silico pharmacokinetics analysis, including Absorption, Distribution, Metabolism, Excretion, and Toxicity (ADMET) studies, were conducted using the SwissADME online server [20] (http://www.swissadme.ch, accessed in 15 June 2024).

### 2.12. Image Acquisition

For phase-contrast and epifluorescence microscopy, we used the Leica DM IL LED microscope, equipped with I3 S, N2.1 S, and Y5 filter systems (Leica Microsystems AG, Wetzlar, Germany). High-resolution images (1296 × 966 pixels) were captured using the Leica DFC3000 G CCD Microscope Camera (Leica Microsystems, Wetzlar, Germany) controlled by Leica LAS X software (Leica Microsystems, Wetzlar, Germany, Version: 3.7.2.22383) (20×, NA 0.4). Immunocytochemistry images were taken with an Olympus BX53^®^ polarizing light microscope assembled with an Olympus DP72^®^ camera at 1296 × 966 pixels, controlled using cellSens™ (Olympus Entry, Inc., 2020, Version 1.30) (100×, NA 1.30) (Center Valley, PA, USA). Image processing was performed with Adobe Photoshop 7.0. Cell and puncta quantifications were carried out using ImageJ software (version 1.49, National Institutes of Health, Bethesda, MD, USA) with the cell counter plugin.

### 2.13. Statistical Analysis

Statistical analyses were executed using GraphPad Prism version 8.0 (San Diego, CA, USA). We employed Student’s *t*-test or one-way ANOVA for group comparisons, followed by Duncan’s multiple comparison tests for post hoc analysis. Statistical significance was defined as *p*-values < 0.05. Data were presented as the standard error of the mean (S.E.M.) from three independent experiments, ensuring the robustness and reproducibility of the results.

## 3. Results

### 3.1. Phytochemical Profiling of the FVSE

#### 3.1.1. GC-MS Analysis

**Figure 1:**
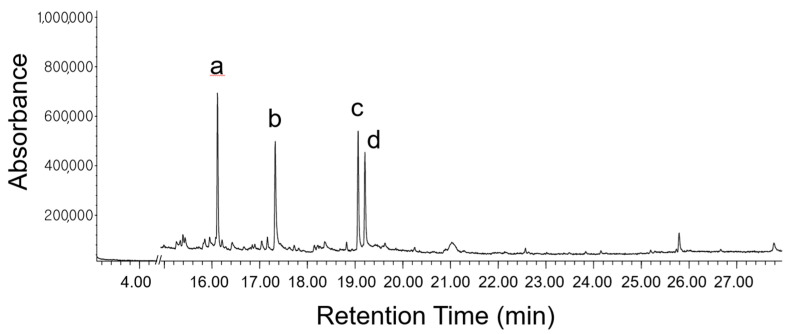
The GC-MS profile of FVSE. The prominent peaks are identified as anethole (a), 1-(4-methoxyphenyl)-2-propanone (b), ethoxydimethylphenylsilane (c), and para-anisaldehyde diethyl acetal (d).

The GC of the FVSE revealed 41 identifiable peaks (Figure 1; the full-scale chromatogram is shown in Supplementary Figure S1), and the chemical identities of peaks were determined by screening the NIST12.0 spectral library (https://www.nist.gov/pml/atomic-spectra-database, accessed in 15 June 2024) using the chemical’s fragmentation patterns in the subsequent MS (Table 1).

#### 3.1.2. The Identities of the Prominent Components

**Figure 2:**
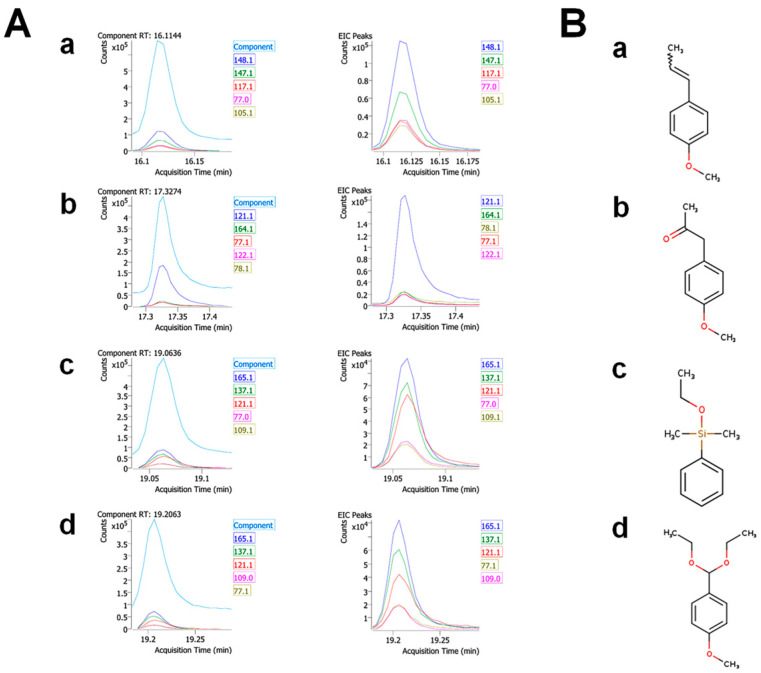
Identities of the four prominent components in *Foeniculum vulgare* Mill. seed ethanol extract (FVSE). (**A**) MS fragmentation patterns. (**B**) Molecular structures. (**a**) Anethole, (**b**) 1-(4-methoxyphenyl)-2-propanone, (**c**) ethoxydimethylphenyl-silane, and (**d**) para-anisaldehyde diethyl acetal. See Supplementary Figure S2 for more extensive data.

The MS fragmentation patterns of the prominent GC components are shown in Figure 2A with extracted ion chromatogram (EIC) peaks and component retention time (RTs). Screening the NIST12.0 spectral library identified the compounds as anethole, 1-(4-methoxyphenyl)-2-propanone, ethoxydimethylphenylsilane, and para-anisaldehyde diethyl acetal (Figure 2B and Supplementary Figure S2). These four prominent components are listed in Table 2 with match factors to a reference spectrum in the database, where anethole shows an almost perfect score of 99. Referring to the percentage values of peak areas, the four compounds apparently comprised 64.4% of the total FVSE phytochemicals.

We further confirmed the identity of the ‘anethole’ peak using an anethole standard with High-Performance Liquid Chromatography (HPLC) and Gas Chromatography–Flame Ionization Detection (GC-FID), which is highly sensitive to organic compounds. First, the retention time of a prominent peak in the HPLC chromatogram of the FVSE exactly matched that of the anethole standard (Supplementary Figure S3). Although the identity of the peak in the FVSE chromatogram is not definitively known, its prominence strongly indicates that it is anethole. Second, the retention time of the ‘anethole’ peak in the FVSE sample exactly matched that of the anethole standard in the GC-FID chromatograms (Supplementary Figure S4), a predominant detection method in GC for identifying and measuring organic compounds. These results provide solid evidence for the identity of the ‘anethole’ peak in GC-MS.

### 3.2. Druggability and Pharmacokinetic Properties of the FVSE’s Main Compounds

#### 3.2.1. Absorption, Distribution, Metabolism, Excretion, and Toxicity (ADMET) Analysis

ADMET predictions serve as an initial step in the drug development pipeline. The use of in silico ADMET models is well-established in early-stage drug discovery to screen compounds, prioritize candidates, and predict potential pharmacokinetic and toxicity profiles, thereby reducing the number of compounds that need to be tested in vitro and in vivo [40,41,42,43].

As a first step, the SMILES (Simplified Molecular Input Line Entry System) notation of the compounds under study was obtained from the PubChem database (https://pubchem.ncbi.nlm.nih.gov/, accessed in 15 June 2024). The drugability and various physicochemical properties of the primary compounds found in FVSE (anethole, 1-(4-methoxyphenyl)-2-propanone, para-anisaldehyde diethyl acetal, and ethoxydimethylphenyl-silane) were evaluated based on their Absorption, Distribution, Metabolism, Excretion, and Toxicity (ADMET) characteristics [14]. The reported properties include rotatable bonds, H-bond acceptors, H-bond donors, topological polar surface area (TPSA, Å^2^), lipophilicity (Moriguchi Log P, MLogP), bioavailability score, violation of Lipinski’s Rule of Five, and gastrointestinal (GI) absorption (Table 3).

#### 3.2.2. Components Boiled-Egg Model and Radar Plot Analysis

**Figure 3:**
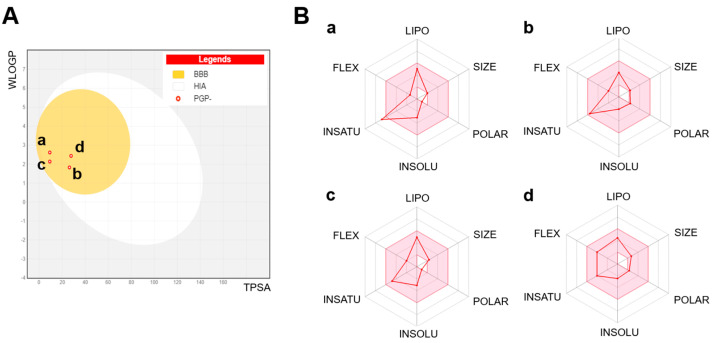
Pharmacokinetic properties generated with the SwissADME web tool. (**A**) A boiled-egg model. Wildman–Crippen LogP (WLOGP). Topological polar surface area (TPSA). (**B**) Radar plots. (**a**) Anethole, (**b**) 1-(4-methoxyphenyl)-2-propanone, (**c**) ethoxydimethylphenyl-silane, and (**d**) para-anisaldehyde diethyl acetal. Lipophilicity (LIPO), SIZE (MW, g/mol), polarity (POLAR), insolubility (INSOLU), instauration (INSATU), and flexibility (FLEX).

The boiled-egg model and radar plot analysis were employed to evaluate the pharmacokinetic properties of the four primary compounds. All four compounds were located in the yellow area of the boiled-egg model (Figure 3A), indicating a high likelihood of blood–brain barrier (BBB) penetration and strong oral bioavailability [44]. The radar plots further revealed that none of the compounds violated Lipinski’s Rule of Five (MW ≤ 500 g/mol; hydrogen bond donors ≤ 5; hydrogen bond acceptors ≤ 10) [15,16] and lacked significant Cytochrome P450 enzyme inhibition (Figure 3B), suggesting their suitability as drug candidates.

Notably, anethole displayed high lipophilicity (LIPO) and very low polarity (POLAR) and flexibility (FLEX; number of rotatable bonds: 2, number of H-bond acceptors: 1, and number of H-bond donors: 0), which are advantageous for membrane permeability and target protein interaction [45]. Overall, these properties suggest that anethole could be a promising candidate for new cardioprotective treatments due to its robust ADME properties and favorable drug-likeness profile.

#### 3.2.3. Consideration for Potential Nutrients

Next, we referred to FooDB (https://www.foodb.ca/), the world’s largest and most comprehensive resource on food constituents, chemistry, and biology, to consider the potential for nutrients. Ethoxydimethylphenylsilane and para-anisaldehyde diethyl acetal were not identified as food or nutrient compounds in FooDB (searched on 20 June 2024). 1-(4-methoxyphenyl)-2-propanone was listed under ‘foods’ such as cattle (beef and veal), chocolate, etc., but not under ‘nutrients’. Instead, this compound is primarily known as an intermediate in the synthesis of various pharmaceuticals and chemicals, such as para-methoxyamphetamine and para-methoxy-N-methylamphetamine [46,47]. In contrast, anethole is a prominent component of the essential oils from plants such as anise and fennel [13,48] and used as a flavoring substance in seasoning and confectionery applications. The United States Food and Drug Administration (FDA-US) has certified its safety. These facts strongly indicate its excellent potential for both nutritional and medicinal applications.

### 3.3. Cardiomyocyte-Protective Activities of FVSE and Its Prominent Component Anethole

#### 3.3.1. Optimization of FVSE and Anethole Concentration

**Figure 4:**
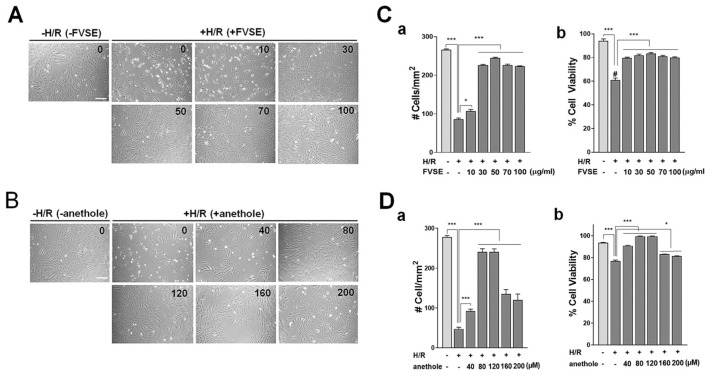
Effects of FVSE and anethole on the viability of H9C2 cells under H/R treatment. (**A**,**B**) Representative images showing a marked increase in cell numbers in H/R treatment. Scale bar: 50 µm. (**Ca**,**Da**) The numbers of all attached cells. (**Cb**,**Db**) The proportions of viable cells among attached cells, assessed by trypan blue exclusion assays. All statistics are expressed as means ± SEM. # number, * *p* < 0.05, *** *p* < 0.001 (ANOVA).

We established the optimal concentration of the FVSE and anethole for cardiomyocyte-protective effects in H9C2 cells following hypoxia/reoxygenation (H/R) stress. For this purpose, cells were grown in varying concentrations of FVSE (0–100 µg/mL) and anethole (0–200 µM) for 16 h before H/R shock. Usually, without a protective agent, H/R treatment induces significant damage to the cells, causing the dead cells to detach from the culture substrate. Therefore, the number of cells remaining attached is important to assess the effects of FVSE and anethole. For this reason, we initially counted the total cell numbers on the culture substrate, regardless of viability. Typical images of cultures are shown in Figure 4A,B, which demonstrate a significant increase in the number of cells in a dose-dependent manner in cultures treated with FVSE (Figure 4A) or anethole (Figure 4B). Statistical analysis revealed that H/R treatment reduced the cell number by 67.9% when FVSE was not present (Figure 4(Ca)). This reduction was recovered up to 15% in a FVSE dose-dependent manner, with an optimal concentration of 50 µg/mL. When live cells were differentiated from dead ones by staining with 0.4% (*w*/*v*) trypan blue, a significant increase in live cell numbers was observed in a dose-dependent manner, with the highest increase at 50 µg/mL in FVSE-treated cultures (Figure 4(Cb)). Anethole exhibited similar effects on the number of cells attached to the substrate (Figure 4(Da)) and the proportion of live cells (Figure 4(Db)), with an optimal positive effect at 80 µM. Therefore, we used 50 µg/mL of FVSE and 80 µM of anethole in subsequent experiments.

#### 3.3.2. FVSE and Anethole Suppress H/R-Induced ROS Generation

**Figure 5:**
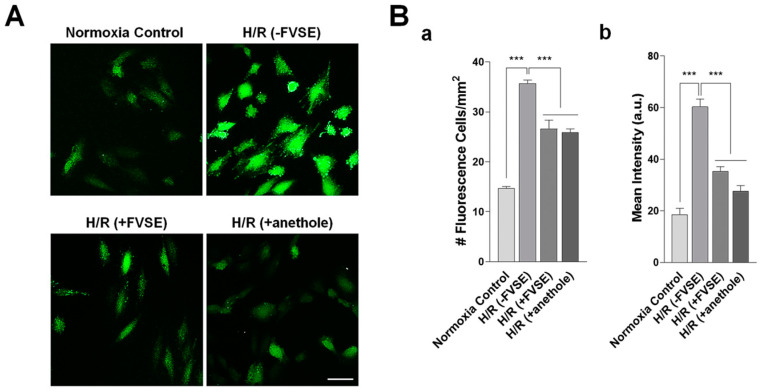
FVSE and anethole suppress ROS Production. Cells were grown and H/R-treated, as described in Figure 4, and stained with DCFDA. (**A**) Representative images showing ROS-producing cells. Scale bar: 50 µm. (**B**) Measurement of ROS-positive cells (**a**) and the relative intensity (arbitrary units, a.u.) (**b**). Bars represent the mean ± SEM (*n* = 3, ~200–300 cells per group). *** *p* < 0.001 (ANOVA).

Given that H/R results in the excessive production of ROS, which imposes oxidative stress on cells, we investigated whether FVSE and anethole could attenuate ROS generation. H9C2 cells were exposed to H/R, and the production of ROS was quantitatively analyzed using DCFDA staining (Figure 5A). In the absence of FVSE ([(−FVSE) H/R] group), the number of ROS-positive cells increased by 2.4-fold (*p* < 0.001). In contrast, pre-treatment with FVSE or anethole ([(+FVSE) H/R] or [(+anethole) H/R] group, respectively) reduced the number of ROS-positive cells by 25.5% (*p* < 0.001) and 27.4% (*p* < 0.001), respectively. In addition to the number of ROS-positive cells, ROS intensity was also measured. In the absence of FVSE [(−FVSE) H/R] group), the intensity of ROS-positive cells increased by 3.26-fold (*p* < 0.001) compared to a control that did not receive H/R shock [(−FVSE) no H/R] group) (Figure 5B). Pre-treatment with FVSE or anethole [(+FVSE) H/R] or [(+anethole) H/R] group, respectively) reduced the ROS intensity by 41.5% and 44.3% (*p* < 0.001), respectively.

#### 3.3.3. FVSE and Anethole Decrease DNA Damage

**Figure 6:**
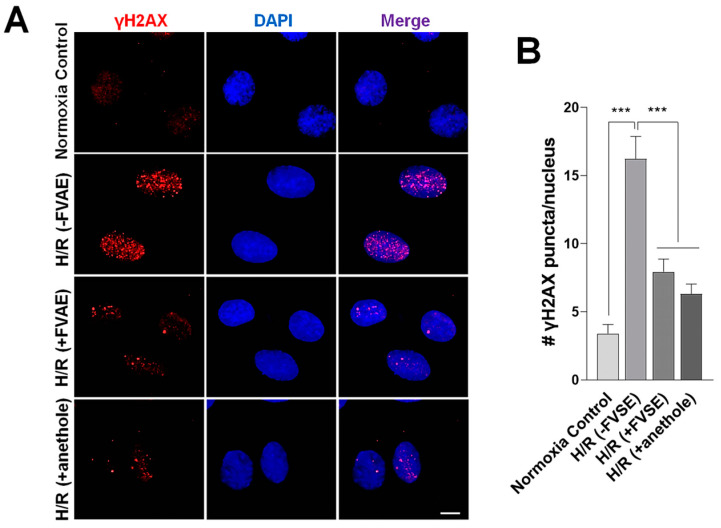
FVSE and anethole mitigate double-strand DNA breakage in H/R. Cells were grown and subjected to H/R shock, as described in Figure 4, then immunostained with an anti-phospho-H2AX antibody and stained with DAPI to reveal nuclei. (**A**) Representative images showing phospho-H2AX puncta. Scale bar: 10 µm. (**B**) Statistical analysis. Bars represent means ± SEM (n = 30 nuclei). *** *p* < 0.001 (ANOVA).

Hypoxia leads to apoptosis, characterized by chromatin condensation, DNA fragmentation, the release of nuclear proteins, cytoplasmic shrinking, and membrane blebbing [49]. When a DNA double-strand break occurs, the H2AX histone is phosphorylated at a specific serine residue (Ser139) at the site of the break, resulting in the formation of γ-H2AX (phosphorylated H2AX). To explore the protective effects of FVSE and anethole against double-stranded DNA breaks under H/R conditions, H9C2 cells were pre-treated with FVSE or anethole, subjected to H/R, and subsequently immunostained with an anti-phospho γ-H2AX antibody (Figure 6A). Quantitative analysis revealed a significant 4.78-fold increase in phospho γ-H2AX immunopuncta per nucleus in the [(−FVSE) H/R] group (Figure 6B) compared to H/R-untreated cells. In contrast, pre-treatment with FVSE or anethole substantially reduced the number of puncta by 51.1% or 61.1% (both *p* < 0.001), respectively, indicating the mitigating effects of FVSE and anethole on H/R-induced DNA breaks.

#### 3.3.4. FVSE and Anethole Prevent Nuclear Condensation

**Figure 7:**
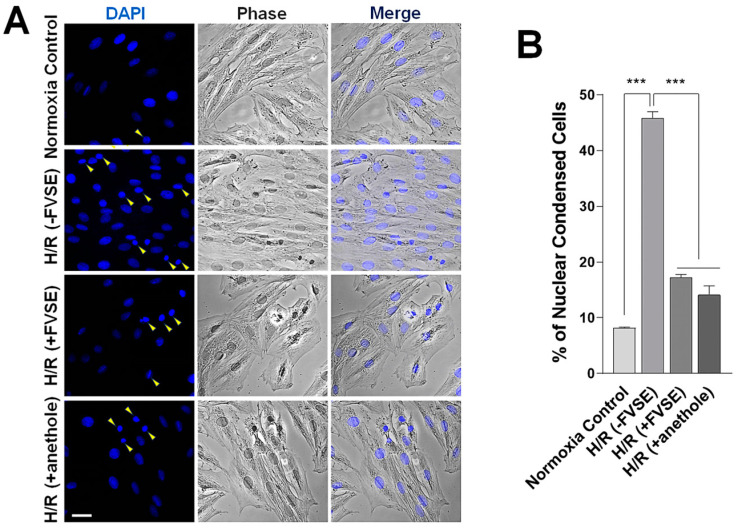
FVSE and anethole prevent nuclear condensation in H/R. H9C2 cells were grown and subjected to H/R shock, as described in Figure 4, then stained with DAPI to reveal nuclei. The condensed nuclei are marked by yellow arrowheads. (**A**) Representative images showing cells with condensed nuclei. Scale bar: 50 µm. (**B**) Statistical analysis. Bars represent means ± SEM (n = ~200–300 nuclei, 3 replicates). *** *p* < 0.001 (ANOVA).

We explored the protective effects of FVSE and anethole on nuclear condensation under H/R conditions. Nuclear shapes were monitored by DAPI staining (Figure 7A). Under normoxia, 8.15% of cells showed condensed nuclei (Figure 7B). This percentage significantly increased by 5.62-fold in the absence of FVSE [(−FVSE) H/R] group). This increase was reduced by 62.6% or 69.3% (both *p* < 0.001) in the presence of FVSE or anethole, respectively, [(+FVSE) H/R] or [(+anethole) H/R] group, indicating that FVSE and anethole effectively prevented nuclear condensation under H/R conditions.

#### 3.3.5. FVSE and Anethole Preserve ΔΨm

**Figure 8:**
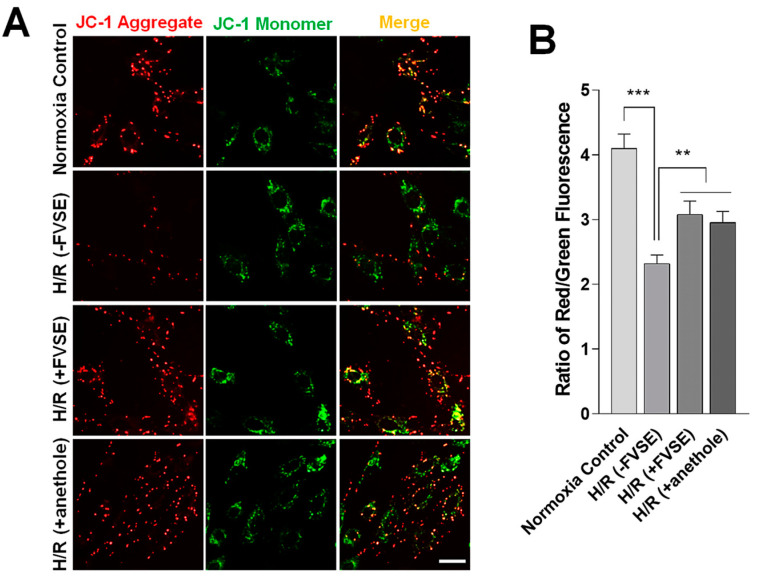
FVSE and anethole prevent ΔΨm dissipation in H/R. H9C2 cells were grown and subjected to H/R shock, as described in Figure 4, and ΔΨm was determined by JC-1 staining. (**A**) Representative images showing red and green fluorescence. Scale bar: 50 μm. (**B**) Statistical analysis. Bars represent means ± SEM (n = ~200–300 spots). ** *p* < 0.01, *** *p* < 0.001 (ANOVA).

JC-1 is a ΔΨm-sensitive dye widely used to assess mitochondrial function [18]. JC-1 monomers, indicative of dysfunctional mitochondria in a depolarized or de-energized state, emit green fluorescence, whereas J-aggregates, indicative of healthy, hyperpolarized, or energized mitochondria, emit orange–red fluorescence (Figure 8A). We measured the red/green fluorescence ratios to evaluate mitochondrial health. This ratio was 4.101 a.u. in normoxia, but decreased to 2.320 (*p* < 0.001) in the [H/R (−FVSE)] group (Figure 8B). Pre-treatment with FVSE [H/R (+FVSE)] or anethole [H/R (+anethole)] reversed this value to 3.079 or 2.951 (both *p* < 0.01), respectively (Figure 8B). These results indicate that FVSE and anethole significantly (*p* < 0.01) attenuate the loss of ΔΨm in H9C2 cells under H/R conditions.

## 4. Discussion

Cardiovascular diseases (CVDs) are the leading cause of death worldwide, prompting extensive research into discovering new therapeutic agents. Natural products have been recognized as potential sources for novel cardioprotective agents. Purified extracts and active compounds from plants and herbs are increasingly incorporated into modern medical systems to address CVDs. In this study, we report that the ethanol extracts of F. vulgare seeds (FVSEs) and the prominent component, anethole, protect against cellular damage in the H9C2 cardiomyocyte hypoxia/reoxygenation (H/R) experimental model. Specifically, FVSE and anethole were found to suppress H/R-induced reactive oxygen species (ROS) generation, DNA damage, nuclear condensation, and the dissipation of mitochondrial membrane potential (ΔΨm).

*F. vulgare*, commonly known as fennel, is renowned for its rich phytochemical composition, which includes flavonoids, phenolic compounds, and essential oils, exhibiting a spectrum of bioactivities, including antioxidative properties relevant to hypoxia-related cardiac damage [13]. After initially finding the mitigating effect of FVSE in H/R, we employed a two-pronged approach that integrates an in vitro H/R experimental model and network pharmacology analysis to further explore its cardioprotective mechanisms. To identify the active phytochemicals in FVSE, we performed GC-MS analysis, which revealed four prominent components: anethole, 1-(4-methoxyphenyl)-2-propanone, para-anisaldehyde diethyl acetal, and ethoxydimethylphenyl-silane. These comprised 64.4% of the total extracts (see Table 2 for each component).

The druggability and several pharmacokinetic properties of these prominent compounds were assessed based on their ADMET attributes [14]. Bioavailability, which mainly depends on the physicochemical characteristics of molecular size (SIZE) and lipophilicity (LIPO), was also evaluated. In the boiled-egg model, all four components were positioned within the yolk region, suggesting a high probability of blood–brain barrier (BBB) penetration and strong oral bioavailability [44]. Radar plots show that anethole exhibits high lipophilicity and flexibility with very low polarity and rigidity, which are favorable for membrane permeability and target interaction [45]. Indeed, it is orally bioavailable [50], and experimental studies on rats and mice showed that anethole is completely absorbed after oral administration, and eliminated within 48–72 h, and the major routes are renal, pulmonary, and fecal excretion (Committee of Experts on Cosmetic Products, 2008).

Anethole satisfies Lipinski’s Rule of Five (molecular weight < 500; octanol-water partition coefficient (Log P) < 5; hydrogen bond donors < 5; hydrogen bond acceptors < 10; topological polar surface area (TPSA) < 140 Å^2^) and exhibits favorable ADMET properties [51]. Anethole shows high druggability with a low molecular weight (148.2 g/mol), minimal rotatable bonds (2), and the highest bioavailability score (0.55) (Table 2). Lower-molecular-weight compounds often correlate with better absorption and distribution due to increased permeability and reduced steric hindrance. Molecules with fewer rotatable bonds enhance pharmacokinetic properties [14]. Lipinski’s rule [15,16] suggests that compounds with more than five HBDs and 10 HBAs exhibit poor membrane permeability, affecting absorption significantly. Anethole has one H-bond acceptor and no H-bond donors, favoring membrane permeability [52]. Compounds with moderate lipophilicity (Log P 1–3) balance solubility in lipid membranes and aqueous environments, ensuring optimal bioavailability [53,54]. Anethole’s LogP of 2.67 suggests moderate lipophilicity, ideal for oral bioavailability. TPSA, which measures the polar surface area of a molecule, indicates that lower values correlate with better permeability. A TPSA under 140 Å^2^ is desirable for oral bioavailability [55]. Anethole’s TPSA of 9.23 Å^2^ and bioavailability score of 0.55 indicate good membrane permeability and oral bioavailability, underscoring its potential as a bioavailable drug candidate.

Next, we referred to FooDB (https://www.foodb.ca, searched on 20 June 2024), the world’s largest and most comprehensive resource on food constituents, chemistry, and biology, to consider potential nutrients and medicinal compounds. Ethoxydimethylphenylsilane and para-anisaldehyde dimethyl acetal were not identified as food or drug components in our search for compounds, food, and nutrients in FooDB (searched on 20 June 2024). 1-(4-methoxyphenyl)-2-propanone was found in small amounts (1.000–6.500 mg/100 g) in foods such as cattle (beef and veal), chocolate, piki bread, wheat bread, and white bread (https://foodb.ca/compounds/FDB010873, searched on 20 June 2024). It has also been detected, but not quantified, in fennel and other herbs and spices [13]. However, it was not listed under ‘nutrients’ in FooDB. Instead, this compound is primarily known as an intermediate in the synthesis of various pharmaceuticals and chemicals, like para-methoxyamphetamine and para-methoxy-N-methylamphetamine, both of which are structurally related to amphetamine but are not commonly used as medications on their own [46,56,57]. In contrast, anethole is a prominent component of the essential oils from plants, such as anise and fennel [13,48]. It significantly contributes to the odor and flavor of anise and fennel (both in the botanical family Apiaceae), anise myrtle (Myrtaceae), licorice (Fabaceae), magnolia blossoms, and star anise (Schisandraceae). Anethole is an organic compound widely used as a flavoring substance for seasoning and confectionery, such as German Lebkuchen, oral hygiene products, and in small quantities in natural berry flavors [58]. It is used in both the food and pharmaceutical industries, and the FDA-US has certified its safety [59]. Experimentally, anethole has shown no toxicity at low doses [60] and is considered non-genotoxic and non-carcinogenic, making it quite safe [61,62].

Anethole bears a structural resemblance to catecholamines, like dopamine, and may displace dopamine from its receptors, thereby disinhibiting prolactin secretion, which may be responsible for its galactagogue effects [63]. Anethole also exhibits estrogenic activity [30]. Additionally, anethole has several medical applications, primarily due to its anti-inflammatory, antioxidant, and antimicrobial properties. In the USA, anethole is generally recognized as safe (GRAS). In the present study, we confirm the efficacy of FVSE and anethole in ameliorating cardiomyocyte damage under hypoxic shock conditions. Considering its pharmacokinetic properties, toxicity, nutritional and medicinal applications, along with our experimental findings, anethole stands out as an excellent target phytochemical for developing drugs for diseases, including CVDs.

## 5. Conclusions

Among the four prominent phytochemicals in the *F. vulgare* ethanol extracts (FVSEs) [anethole, 1-(4-methoxyphenyl)-2-propanone, ethoxydimethylphenylsilane, and para-anisaldehyde diethyl acetal], anethole stands out due to its potential nutritional and pharmacokinetic properties as assessed by ADMET analysis, including bioavailability, lipophilicity, flexibility, and compliance with Lipinski’s Rule of Five. Both FVSE and its prominent component anethole have been shown to suppress hypoxia/reoxygenation (H/R)-induced reactive oxygen species (ROS) generation, DNA double-strand break damage, nuclear condensation, and dissipation of mitochondrial membrane potential (ΔΨm). Hypoxia adversely affects the integrity of cardiomyocytes, playing a critical role in the development of cardiovascular diseases (CVDs). Traditional treatments for CVDs are often expensive and may have undesirable side effects. *F. vulgare* Mill., commonly known as fennel, is a safe, aromatic, medicinal plant and culinary herb that is popular worldwide. Therefore, this study highlights the therapeutic potential of FVSE and its prominent component anethole in the treatment of CVDs, particularly those associated with hypoxia-induced damage.

## Figures and Tables

**Table 1:** Identified phytochemicals in *F. vulgare* seed ethanol extract (FVSE).

No.	RT	Compound Name	CAS No.	% Area	Match Factor	Pharmacological Activities (Ref.)
1	12.9749	Phenol	108-95-2	3.00	90.6	Antiseptic [21]
2	13.1057	2-Hydroxy-gamma-butyrolactone	19444-84-9	1.43	81.3	Antimicrobial [22]
3	13.516	Furan, 2-butyltetrahydro-	1004-29-1	1.36	65.4	No activities
4	13.6349	Cyclopropanemethanol, alpha-butyl-	4379-16-2	0.69	64.7	No activities
5	13.9441	Phenol, 2-methoxy-	1990-05-01	0.44	76.1	Antioxidant [23]
6	14.9906	5-Methyl-1R,3-trans-cyclohexanediol	549277	0.25	61.3	No activities
7	15.2522	4-Vinylphenol	2628-17-3	0.69	67.6	Antimicrobial [24]
8	15.3355	2-Oxabicyclo[2.2.2]octan-6-ol, 1,3,3-trimethyl-	5418-86-0	0.59	77.1	No activities
9	15.389	Methane, tris(methylthio)-	18679-48-6	1.07	83.4	No activities
10	15.8528	2-Hydroxymethyl-2-methylbrendane	584818	0.69	70.7	No activities
11	15.9479	9-Decyn-1-ol	17643-36-6	1.00	75.4	Antifungal [25]
12	16.1144	Anethole	104-46-1	17.84	99	Anti-inflammatory, Antioxidant, anticancer, neuroprotective, cardio-protective, gastro-protective, antifungal, and antispasmodic [26,27,28]
13	16.2095	Cyclohexane, 2-ethenyl-1,1-dimethyl-3-methylene-	95452-08-7	0.95	71.9	No activities
14	16.4236	Phenol, 5-ethenyl-2-methoxy-	621-58-9	1.45	83.9	Antioxidant [29]
15	16.6674	2,5-Dimethylcyclohexanol	3809-32-3	0.46	62.6	No activities
16	16.8458	1,2-Cyclohexanediol, 1-methyl-4-(1-methylethenyl)-	1946-00-5	0.38	78.9	No activities
17	16.8993	Bicyclo(3.1.1)heptane-2,3-diol, 2,6,6-trimethyl-	53404-49-2	0.37	63.8	Antioxidant [30]
18	17.042	Benzene, 1-methoxy-4-(1-methylpropyl)-	4917-90-2	1.15	80.7	No activities
19	17.1609	3(2H)-Furanone, 4-methoxy-2,5-dimethyl-	4077-47-8	1.37	69.4	No activities
20	17.3274	2-Propanone, 1-(4-methoxyphenyl)-	122-84-9	17.32	89	Neuroprotective and cardioprotective [31,32]
21	17.7198	1,3,3-Trimethyl-2-oxabicyclo[2.2.2]octane-6,7-endo,endo-diol	56084-15-2	0.66	76.6	No activities
22	17.8209	4-Hydroxybutyl acrylate, TMS	53954667	0.24	59.3	No activities
23	18.1479	Epoxy-linalooloxide	537453	0.76	67.4	Antimicrobial [33]
24	18.2133	2,4,6-Heptanetrione	12285	0.64	47.9	No activities
25	18.2609	1-Propanone, 1-(3-methoxyphenyl)-	37951-49-8	0.09	81.2	No activities
26	18.362	S-(p-Methoxybenzoyl)thiohydroxylamine	35124-66-4	1.23	81.2	No activities
27	18.8198	2,4-Di-tert-butylphenol	96-76-4	1.01	86.3	Antioxidant [34]
28	19.0636	Silane, ethoxydimethylphenyl-	1825-58-7	16.60	83.7	No activities
29	19.2063	para-Anisaldehyde diethyl acetal	75468	12.69	83.2	No activities
30	19.6285	2-Dodecenal, (E)-	20407-84-5	0.58	69.1	Antimicrobial [35]
31	20.2528	1,3-Benzodioxole, 4,5-dimethoxy-6-(2-propenyl)-	484-31-1	0.23	77.8	Antifungal [36]
32	21.0258	1-Hydroxy-1-(4-methoxyphenyl) propane-2-one	15482-29-8	4.78	78.3	Anticencer [37]
33	22.5717	Neophytadiene	504-96-1	0.50	74.3	Anti-inflammatory [38]
34	23.8382	Dibutyl phthalate	84-74-2	0.21	67.8	No activities
35	24.1534	Hexadecanoic acid, ethyl ester	628-97-7	0.49	61.9	No activities
36	25.1939	9-Octadecenoic acid (Z)-, methyl ester	112-62-9	0.43	65.2	No activities
37	25.7409	2-Chloroethyl linoleate	25525-76-2	0.41	70.3	No activities
38	25.7944	Ethyl Oleate	111-62-6	3.30	57.3	No activities
39	26.6685	(2R,3S,5S,6R)-2,5-bis(4-Methoxyphenyl)-3,6-dimethyl-1,4-dioxane-rel-	212516-42-2	0.36	57.3	No activities
40	27.7804	9-Octadecenamide, (Z)-	301-02-0	1.68	80.4	Neuroprotective [39]
41	28.6426	Silane, ethyldimethylphenyl-	17873-23-3	0.45	57	No activities

**Table 2:** Major phytochemicals in *F. vulgare* seed ethanol extract (FVSE).

RT (min)	Compound Name	CAS No.	% of Total	Match Factor
16.1	Anethole	104-46-1	17.8	99
17.3	1-(4-methoxyphenyl)-2-propanone	122-84-9	17.3	89
19.1	Ethoxydimethylphenyl-silane	1825-58-7	16.6	83.7
19.2	Para-Anisaldehyde diethyl acetal	75468	12.7	83.2

**Table 3:** Predicted physicochemical, lipophilicity, pharmacokinetics, drug-likeness, and medicinal properties of FVSE components using SwissADME.

Compound Name	Physicochemical Properties	Lipophilicity (MLogP)	Pharmacokinetics	Drug-Likeness	Medicinal Score
Anethole	MW: 148.2 Rotatable bonds: 2 H-bond donors: 0 H-bond acceptors: 1 TPSA: 9.23 Å^2^	2.67	BBB permeation: Yes GI absorption: High CYP inhibition: Yes on CYP1A2	Lipinski’s rule: Compliant	Predicted bioavailability: 0.55 P-gp substrate: No
1-(4-Methoxyphenyl)-2-propanone	MW: 180.32, Rotatable bonds: 3 H-bond donors: 0 H-bond acceptors: 1 TPSA: 9.23 Å^2^	1.74	BBB permeation: Yes GI absorption: High CYP inhibition: Yes on CYP1A2 and CYP3A4	Lipinski’s rule: Compliant	Predicted bioavailability: 0.55 P-gp substrate: No
Ethoxydimethylphenylsilane	MW: 210.27 Rotatable bonds: 6 H-bond donors: 0 H-bond acceptors: 3 TPSA: 27.67 Å^2^	2.49	BBB permeation: Yes GI absorption: High CYP inhibition: Yes on CYP1A2 and CYP3A4	Lipinski’s rule: Compliant	Predicted bioavailability: 0.55 P-gp substrate: No
Para-Anisaldehyde Diethyl Acetal	MW: 164.2 Rotatable bonds: 3 H-bond donors: 0 H-bond acceptors: 2 TPSA: 26.3 Å^2^	2.20	BBB permeation: Yes GI absorption: High CYP inhibition: Yes on CYP1A2	Lipinski’s rule: Compliant	Predicted bioavailability: 0.55 P-gp substrate: No

## Data Availability

The data that support the findings of this study are available from the corresponding authors upon reasonable request.

## Supplementary Materials

The following supporting information can be downloaded at: https://www.mdpi.com/article/10.3390/antiox13101161/s1.
