# Supplementary material for: Protective Effects of Anethole in Foeniculum vulgare Mill. Seed Ethanol Extract on Hypoxia/Reoxygenation Injury in H9C2 Heart Myoblast Cells
Source: Antioxidants (Basel). 2024 Sep 25;13(10):1161. doi: 10.3390/antiox13101161 (PMC11504384; doi:10.3390/antiox13101161)
Supplement: Supplementary file 1 [file antioxidants-13-01161-s001.zip › Supplementary Figure Legends-Uploaded.pdf]

Supplementary Figure Legends

[Antioxidants] Manuscript ID: antioxidants-3143165

Seo et al.

Supplementary Figure S1. Gas Chromatography-Mass Spectrometry (GC-MS) Profile of *F. vulgare* Mill. Seed Ethanol Extracts (FVSE). The GC-MS analysis was performed as described in section 2.10, "Gas Chromatography-Mass Spectrometry (GC-MS) Analysis" in the Materials and Methods. (A) GC profile of the solvent DMSO. (B) GC profile of FVSE. The prominent peaks were identified by MS as marked phytochemicals.

Supplementary Figure S2. The MS fragmentation patterns and their matches to the NIST 12.0 spectral library. The GC-MS analysis was performed as described in section 2.10, "Gas Chromatography-Mass Spectrometry (GC-MS) Analysis," in the Materials and Methods. The spectrum of fragment ions with specific mass-to-charge ratios (m/z) and their intensities for each phytochemical peak was searched against the NIST (National Institute of Standards and Technology) 12.0 spectral library.

Supplementary Figure S3. Identification of the 'anethole peak' by ultra-high-performance liquid chromatography (U-HPLC). A 20 μL sample of the extract and an anethole standard, dissolved in HPLC-grade methanol and filtered through a 0.2 μm Whatman filter, was injected into an U-HPLC instrument equipped with a photodiode array detector. The sample was separated on a Mightysil reverse-phase C18 column (4.6 mm × 250 mm, 5 μm) using gradient elution with acetonitrile and water at a flow rate of 1 mL/min. The gradient program was as follows: 10% acetonitrile for 0-1 minutes, 50% acetonitrile for 1-8 minutes, 70% acetonitrile for 8-14 minutes, 95% acetonitrile for 14-16 minutes, and 10% acetonitrile for 16-25 minutes. The absorbance of the substrates was monitored at 260 nm, and the relative area (%) of anethole was determined from the peaks observed in the chromatogram. A retention time of 18.4 minutes was observed for both the anethole standard and the corresponding peak in the FVSE extract, confirming the presence of anethole in the sample. The relative area (%) of anethole in the extract was determined to be 10.67%.

Supplementary Figure S4. Identification of the 'anethole peak' by gas chromatography-flame ionization detection (GC-FID). To confirm the identity of the 'anethole peak' marked with a red arrow in FVSE (A), 100,000 ppm of standard trans-anethole was added to the FVSE (B) or used as a standard alone (C), and subjected to GC-FID analysis (Agilent 8890, Agilent Technologies, Santa Clara, CA, USA).
