# Supplementary material for: Protective Effects of Anethole in Foeniculum vulgare Mill. Seed Ethanol Extract on Hypoxia/Reoxygenation Injury in H9C2 Heart Myoblast Cells
Source: Antioxidants (Basel). 2024 Sep 25;13(10):1161. doi: 10.3390/antiox13101161 (PMC11504384; doi:10.3390/antiox13101161)
Supplement: Supplementary file 1 [file antioxidants-13-01161-s001.zip › supplementary1.pptx]

## Slide 1
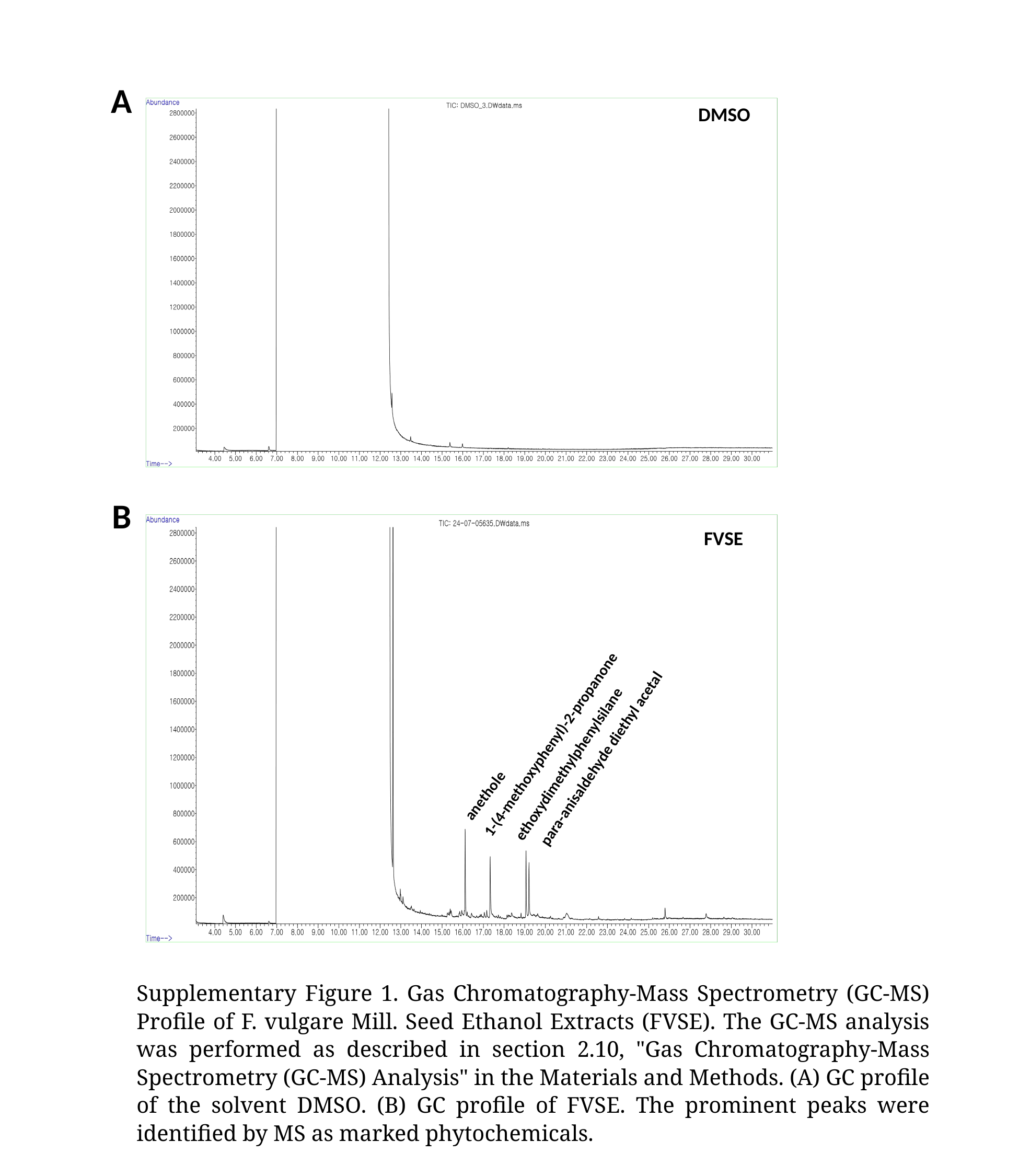

A
DMSO
B
1-(4-methoxyphenyl)-2-propanone
ethoxydimethylphenylsilane
para-anisaldehyde diethyl acetal
anethole
FVSE
Supplementary Figure 1. Gas Chromatography-Mass Spectrometry (GC-MS) Profile of F. vulgare Mill. Seed Ethanol Extracts (FVSE). The GC-MS analysis was performed as described in section 2.10, "Gas Chromatography-Mass Spectrometry (GC-MS) Analysis" in the Materials and Methods. (A) GC profile of the solvent DMSO. (B) GC profile of FVSE. The prominent peaks were identified by MS as marked phytochemicals.
