# Supplementary material for: Protective Effects of Anethole in Foeniculum vulgare Mill. Seed Ethanol Extract on Hypoxia/Reoxygenation Injury in H9C2 Heart Myoblast Cells
Source: Antioxidants (Basel). 2024 Sep 25;13(10):1161. doi: 10.3390/antiox13101161 (PMC11504384; doi:10.3390/antiox13101161)
Supplement: Supplementary file 1 [file antioxidants-13-01161-s001.zip › supplementary2.pptx]

## Slide 1
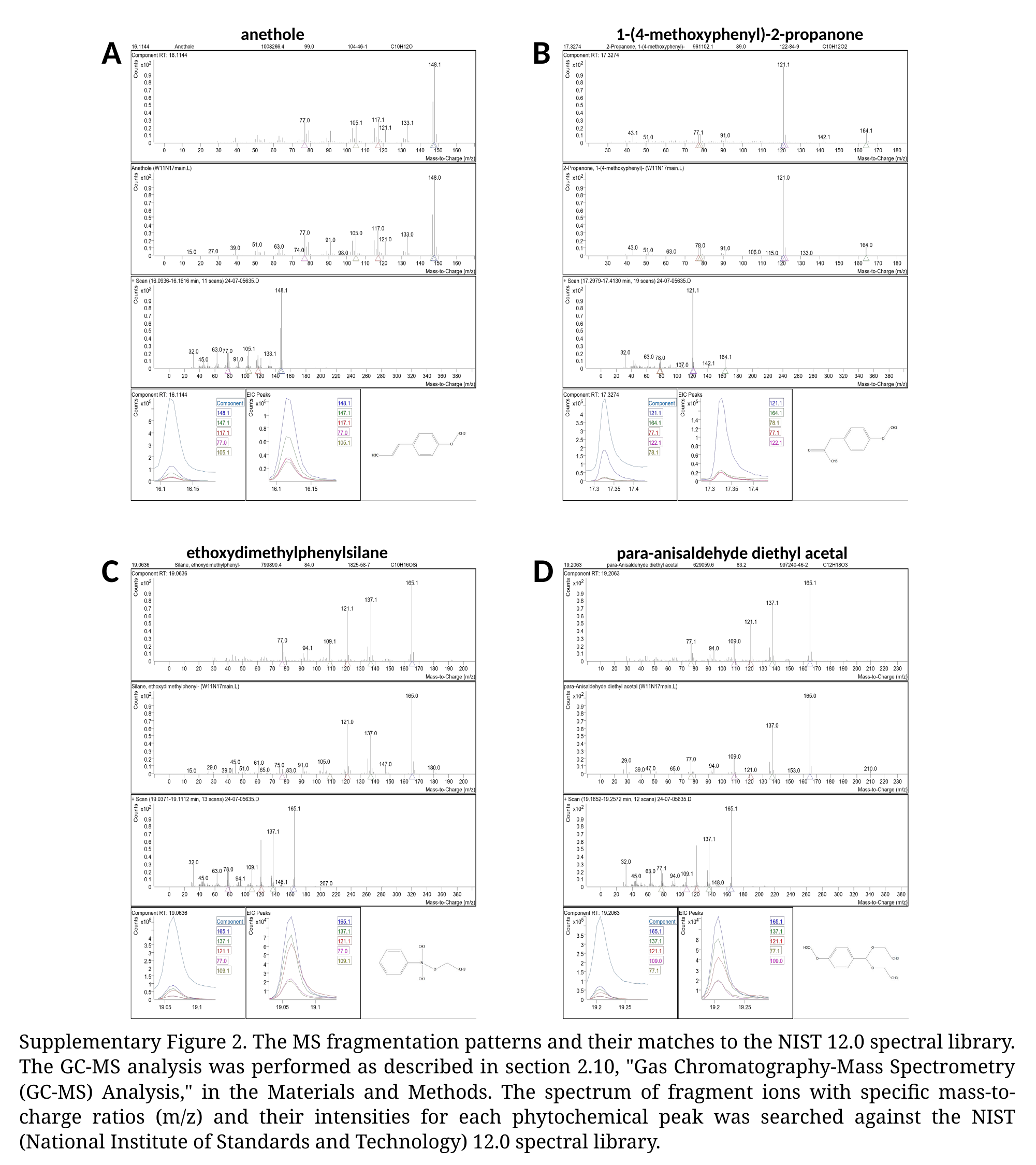

1-(4-methoxyphenyl)-2-propanone
anethole
A
B
1-(4-methoxyphenyl)-2-propanone
anethole
A
B
ethoxydimethylphenylsilane
para-anisaldehyde diethyl acetal
C
D
Supplementary Figure 2. The MS fragmentation patterns and their matches to the NIST 12.0 spectral library. The GC-MS analysis was performed as described in section 2.10, "Gas Chromatography-Mass Spectrometry (GC-MS) Analysis," in the Materials and Methods. The spectrum of fragment ions with specific mass-to-charge ratios (m/z) and their intensities for each phytochemical peak was searched against the NIST (National Institute of Standards and Technology) 12.0 spectral library.
