# Supplementary material for: Protective Effects of Anethole in Foeniculum vulgare Mill. Seed Ethanol Extract on Hypoxia/Reoxygenation Injury in H9C2 Heart Myoblast Cells
Source: Antioxidants (Basel). 2024 Sep 25;13(10):1161. doi: 10.3390/antiox13101161 (PMC11504384; doi:10.3390/antiox13101161)
Supplement: Supplementary file 1 [file antioxidants-13-01161-s001.zip › supplementary3.pptx]

## Slide 1
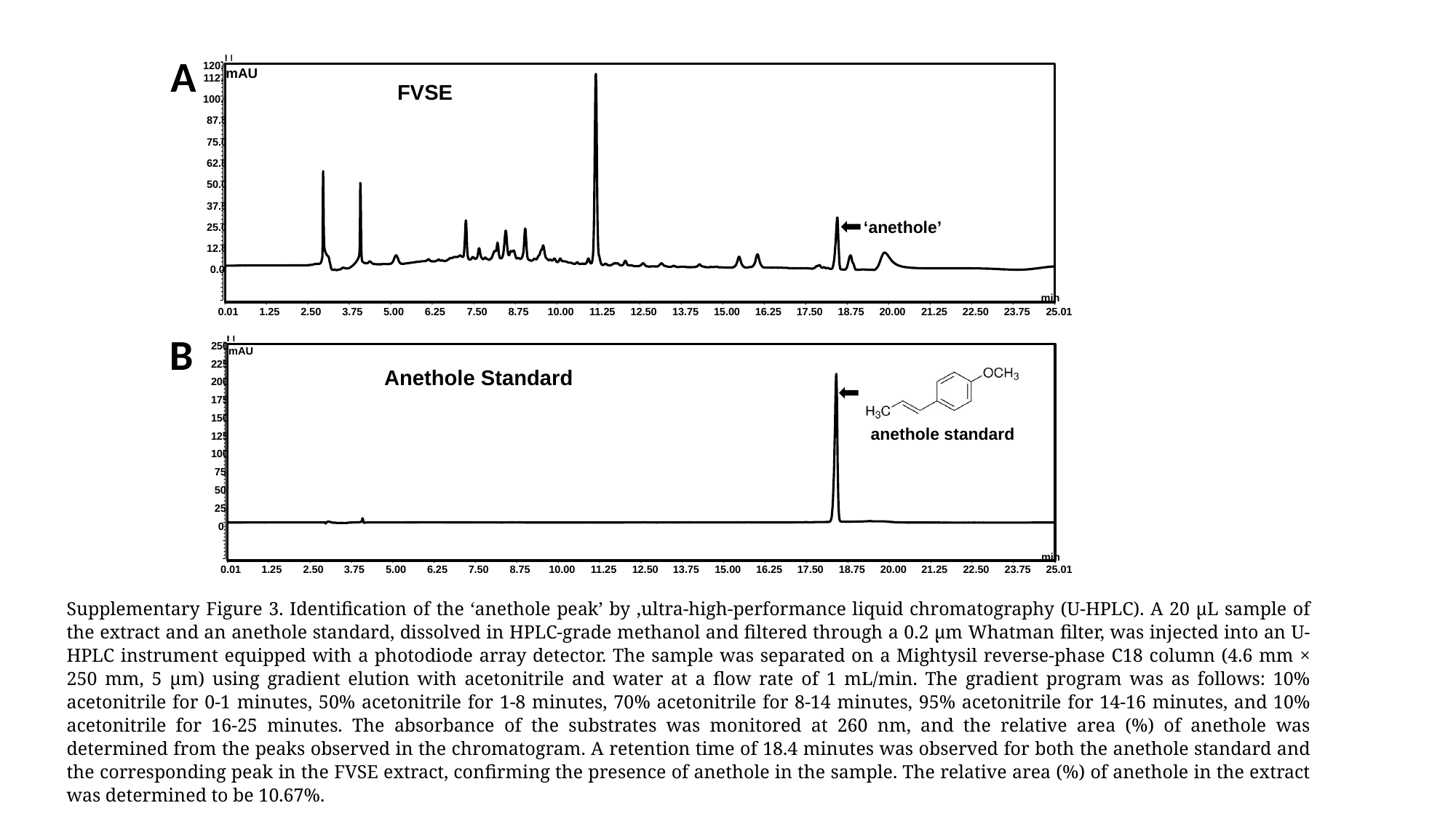

A
120.0
mAU
112.5
100.0
87.5
75.0
62.5
50.0
37.5
25.0
12.5
0.0
min
0.01
1.25
2.50
3.75
5.00
6.25
7.50
8.75
10.00
11.25
12.50
13.75
15.00
16.25
17.50
18.75
20.00
21.25
22.50
23.75
25.01
FVSE
‘anethole’
B
250
225
200
175
150
125
100
75
50
25
0
0.01
1.25
2.50
3.75
5.00
6.25
7.50
8.75
10.00
11.25
12.50
13.75
15.00
16.25
17.50
18.75
20.00
21.25
22.50
23.75
25.01
mAU
Anethole Standard
anethole standard
min
Supplementary Figure 3. Identification of the ‘anethole peak’ by ,ultra-high-performance liquid chromatography (U-HPLC). A 20 μL sample of the extract and an anethole standard, dissolved in HPLC-grade methanol and filtered through a 0.2 μm Whatman filter, was injected into an U-HPLC instrument equipped with a photodiode array detector. The sample was separated on a Mightysil reverse-phase C18 column (4.6 mm × 250 mm, 5 μm) using gradient elution with acetonitrile and water at a flow rate of 1 mL/min. The gradient program was as follows: 10% acetonitrile for 0-1 minutes, 50% acetonitrile for 1-8 minutes, 70% acetonitrile for 8-14 minutes, 95% acetonitrile for 14-16 minutes, and 10% acetonitrile for 16-25 minutes. The absorbance of the substrates was monitored at 260 nm, and the relative area (%) of anethole was determined from the peaks observed in the chromatogram. A retention time of 18.4 minutes was observed for both the anethole standard and the corresponding peak in the FVSE extract, confirming the presence of anethole in the sample. The relative area (%) of anethole in the extract was determined to be 10.67%.
