# Supplementary material for: Protective Effects of Anethole in Foeniculum vulgare Mill. Seed Ethanol Extract on Hypoxia/Reoxygenation Injury in H9C2 Heart Myoblast Cells
Source: Antioxidants (Basel). 2024 Sep 25;13(10):1161. doi: 10.3390/antiox13101161 (PMC11504384; doi:10.3390/antiox13101161)
Supplement: Supplementary file 1 [file antioxidants-13-01161-s001.zip › supplementary4.pptx]

## Slide 1
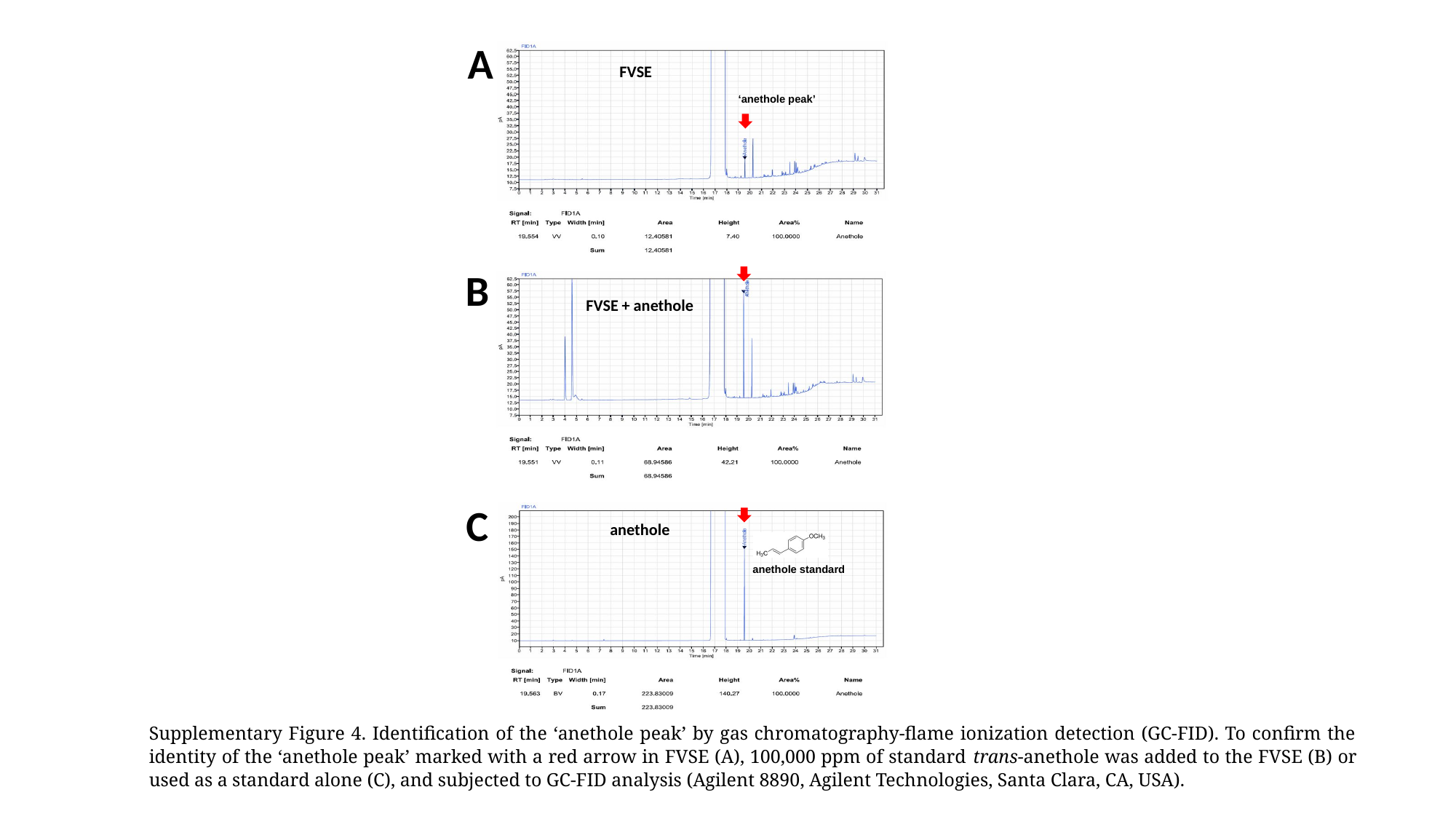

A
FVSE
‘anethole peak’
B
FVSE + anethole
C
anethole
anethole standard
Supplementary Figure 4. Identification of the ‘anethole peak’ by gas chromatography-flame ionization detection (GC-FID). To confirm the identity of the ‘anethole peak’ marked with a red arrow in FVSE (A), 100,000 ppm of standard trans-anethole was added to the FVSE (B) or used as a standard alone (C), and subjected to GC-FID analysis (Agilent 8890, Agilent Technologies, Santa Clara, CA, USA).
